# High expression level of the FTH1 gene is associated with poor prognosis in children with non-M3 acute myeloid leukemia

**DOI:** 10.3389/fonc.2022.1068094

**Published:** 2023-02-01

**Authors:** Junlin Zhang, Liying Liu, Jinshuang Wei, Xiaojing Wu, Jianming Luo, Hongying Wei, Liao Ning, Yunyan He

**Affiliations:** ^1^ First Affiliated Hospital of Guangxi Medical University, Nanning, China; ^2^ The Key Laboratory of Children’s Disease Research in Guangxi’s Colleges and Universities, Education Department of Guangxi Zhuang Autonomous Region, Nanning, China

**Keywords:** AML, WGCNA, FTH1, ferroptosis, prognosis

## Abstract

Acute myelogenous leukemia (AML) is a disease that severely affects the physical health of children. Thus, we aimed to identify biomarkers associated with AML prognosis in children. Using transcriptomics on an mRNA dataset from 27 children with non-M3 AML, we selected genes from among those with the top 5000 median absolute deviation (MAD) values for subsequent analysis which showed that two modules were associated with AML risk groups. Thus, enrichment analysis was performed using genes from these modules. A one-way Cox analysis was performed on a dataset of 149 non-M3 AML patients downloaded from the TCGA. This identified four genes as significant: FTH1, RCC2, ABHD17B, and IRAK1. Through survival analysis, FTH1 was identified as a key gene associated with AML prognosis. We verified the proliferative and regulatory effects of ferroptosis on MOLM-13 and THP-1 cells using Liproxstatin-1 and Erastin respectively by CCK-8 and flow cytometry assays. Furthermore, we assayed expression levels of FTH1 in MOLM-13 and THP-1 cells after induction and inhibition of ferroptosis by real-time quantitative PCR, which showed that upregulated FTH1 expression promoted proliferation and inhibited apoptosis in leukemia cells. In conclusion, high expression of FTH1 promoted proliferation and inhibited apoptosis of leukemic cells through the ferroptosis pathway and is thus a potential risk factor that affects the prognosis of non-M3 AML in children.

## 1 Introduction

Acute myeloid leukemia (AML) is a malignant bone marrow disease (1) that is characterized by clonal expansion as well as stagnated differentiation of immature myeloid cells. About one-fifth of children with acute leukemia have AML, and this incidence is on the rise (2). Although the overall survival rate of children under the age of 19 is about 65% (3), AML contributes to half the mortality rate of childhood leukemia (4). Genetic mutations are the main pathogenic factor (5), in AML, and their identification is thus important in the risk stratification of patients and the determination of appropriate treatment —current treatment is mostly intensive chemotherapy and hematopoietic stem cell transplantation, which both yield poor prognoses. Although there have been meaningful breakthroughs into the genetics of pediatric AML and how it affects the pathophysiological and biological effects on the disease, these are yet to be translated into standard treatment regimens (6).

In the prognostic classification of AML, the survival rate of child in the poor-risk group was actually lower than that of patients in the intermediate and low-risk groups (7, 8). Therefore, the aim of this study was the identification of novel biomarkers for prognostic risk grouping of pediatric AML patients, which possibly facilitate better outcome prediction and individualized treatments. Weighted gene co-expression network analysis (WGCNA) that identifies correlated gene clusters (modules), has been applied to identify biomarkers for ovarian (9) and breast cancers[ (10), demonstrating the feasibility of its use to identify biomarkers for AML.

Ferroptosis, which is a brand of cell death that is not only dependent on intracellular iron but is also morphologically, biochemically, together with genetically distinct from apoptosis, necrosis, as well as autophagy. Ferroptosis plays a crucial regulatory role in the development,along with progression of many diseases, such as tumors, neurological disorders, acute kidney injury, and ischemia/reperfusion. Thus, mitigating disease progression by activating or blocking ferritin production pathways is a possible novel therapeutic strategy for many diseases (11, 12).

In this study, we collected then analyzed with bioinformatics, transcriptome sequencing data from 27 child AML patients and from the TCGA database (https://portal.gdc.cancer.gov/). We identified 60 genes that were potentially associated with risk classification of AML as per WGCNA. Subsequently, we identified FTH1 (Ferritin heavy chain 1) as a key gene associated with survival in AML patients, and confirmed *in vitro*, its effect on the proliferation of leukemic cells.

## 2 Materials and methods

### 2.1 Gene expression data collection

Included in the Collaboration Group of Affiliated Children’s Hospital of Suzhou University, 27 non-M3 AML patients admitted to the First Affiliated Hospital of Guangxi Medical University. Transcriptome sequencing data and clinical information were collected from 27 pediatric non-M3 AML patients admitted to between August 2019 and August 2021. Transcriptome sequencing data and clinical information for 149 children with non-M3-AML were obtained from the TCGA database. The hospital dataset was used for WGCNA to identify candidate key genes, whereas TCGA data were used for survival analysis to further identify prognostic genes. Clinical traits are shown in **Table 1**. Six non-M3 AML patients and six normal people diagnosed and treated in the First Affiliated Hospital of Guangxi Medical University were collected for subsequent experimental verification.

**Table 1:** Demographic and clinical characteristics of AML patients.

Characteristics	Hospital	TCGA
N	27	149
Age, year (median, range)	9.7 (1.5-14.0)	9.3 (0.4-22.6)
Gender (%)		
-Female	13 (48.1)	75 (50.3)
-Male	14 (51.9)	74 (49.7)
WBC		
->100×10^9/^L	3	38
-≤100×10^9/^L	24	111
Median OS (days)	500	1464
Group		
-favorable	5	33
-intermediate	9	57
-poor	13	59
t (8;21)		
-Yes	6	21
-No	21	124
- Unknown	0	4
t (6;9)		
-Yes	1	1
-No	26	144
- Unknown	0	4
t (6;11) (q27; q23)		
-Yes	1	3
-No	26	142
- Unknown	0	4
t (9;11) (p22; q23)		
-Yes	1	13
-No	26	131
- Unknown	0	5
Inv (16)		
-Yes	3	28
-No	24	117
- Unknown	0	4
MLL		
-Yes	4	24
-No	23	121
- Unknown	0	4
WT1 mutation		
-Yes	2	10
-No	25	134
- Unknown	0	5
FLT3-ITD		
-Yes	4	13
-No	23	135
- Unknown	0	1
FAB		
-M0	0	4
-M1	0	15
-M2	5	35
-M4	8	43
-M5	12	29
-M6	0	2
-M7	2	7
-NOS	0	6
- Unknown	0	8

WBC, white blood cell; OS, Overall Survival; FAB, French-American-British cooperative group.

### 2.2 WGCNA

Using median absolute deviation (MAD) values (13). The top 5000 genes were identified from the 27-patient dataset. A scale-free co-expression network was built by WGCNA package in R software, with β set to 14 (scale-free R2 = 0.93) to protect a scale-free network. Next, the adjacency matrix was converted to a topological overlap matrix (TOM) (14) to cluster genes with analogous expression profiles into modules by a mean linkage hierarchical clustering approach. Notably, the minimum number of genes per gene network module (15) was set to 30, and the Dynamic cut tree method algorithm was used to determine the gene network modules.

### 2.3 Identification of candidate biomarkers

Key genes in co-expression networks are those that have high connectivity within network modules and are significantly associated with biological functions. Gene connectivity is measured by the absolute value of the module membership (MM) score, which represents the Pearson correlation coefficient between a particular gene and the module trait value. We selected modules whose gene modules met a p-value less than 0.05, had clinical traits, and calculated the gene significance (GS) scores in absolute values, which indicated the correlation between the genes in these modules and each phenotype (16). Candidate genes were identified by MM and GS scores.

### 2.4 Functional enrichment analysis

On the ground of the protein-protein interactions (PPI) from STRING (https://cn.string-db.org), candidate biomarker genes were constructed in each clinically vital module and visualized using Cytoscape (17). In addition, Gene ontology (GO) enrichment analysis and Kyoto encyclopedia of genes and genomes (KEGG) pathway analysis were performed using R package clusterProfiler.

### 2.5 Determination of the prognostic value of genes

Sixty candidate genes from the 27 leukemia patient dataset, were subjected to univariate Cox regression analysis in the TCGA dataset using the SURVIVAL package on R software, and log-rank p-values as well as hazard ratio (HR) were subsequently calculated. In the end, genes that were statistically significantly associated (*p*<0.05) with prognosis in both datasets were used as biomarkers associated with survival and further investigated using *in vitro* experiments.

### 2.6 Cell culture and drug intervention

MOLM-13 and THP-1 cells were cultured in RPMI 1640 medium, which also containing 10% FBS and 1% penicillin-streptomycin at 37°C along with 5% CO2. *FTH1* was targeted for inhibition of ferroptosis (18) Liproxstatin-1 (1 μM) and Erastin (100 μM) interventions were respectively used as an influential inhibitor and agonist of ferroptosis (19) in MOLM-13 and THP-1 cells. AML cell lines are shown in **Table 2**.

**Table 2:** AML cell line types.

Cell name	Disease	Cytogenetics
THP-1	acute monocytic leukemia	human near-tetraploid karyotype-94 (88–96)<4n>XY/XXY, Y,+1,+3,+6,+6,-8,-13,-19,-22,-22, +2mar, add(1)(p11), del(1)(q42.2) i(2q), del(p21)x2-4, i(7p), der(9)t(9;11)(p22;q23)i(9)(p10)x2,der(11)t(9;11)(p22;q23)x2, add(12)(q24)x1-2, der(13)t(8;13)(p11;p12), add(?18)(q21)- carries t(9;11) associated with AML M5
MOLM-13	acute myeloid leukemia	human hyperdiploid karyotype with 4% polyploidy-51(48-52)<2n>XY, +8, +8, +8,+13,del(8)(p1?p2)?,ins(11;9)(q23;p22p23)

### 2.7 CCK-8 detection

Ninety-six-well plates were inoculated with MOLM-13 and THP-1 cells (3000 cells/well) in RPMI 1640 media supplemented with CCK-8 reagent for 2 h. The optical density at 450 nm (OD450nm) of MOLM-13 and THP-1 cells at 0 h, 12 h, 24 h, 36 h, 48 h, and 60 h after inoculation. All the experiments were repeated four times with similar results.

### 2.8 Cell apoptosis detection by flow cytometry

For the purpose of verify the effect of ferroptosis on apoptosis in leukemia cells, we collected MOLM-13 and THP-1 cells and stained them with the Annexin V-FITC/PI Apoptosis Detection Kit (BD Bioscience, San Jose, CA, USA). Cells were resuspended in 200 µL of 1 × binding buffer at a density of 1 × 106 cells/mL. Then, 5 μL Annexin V-FITC and 5 μL PI were added to the cell suspension and incubated in the dark for 15 min, followed by an analysis of apoptosis using flow cytometry (BD Bioscience, San Jose, CA, USA). All the experiments were repeated three times with similar results.

### 2.9 RNA isolation and qPCR experiment

Six cases of whole blood from non-M3 AML patients and six cases of normal human whole blood and three groups of MOLM-13 and THP-1 cells in normal culture, Liproxstain intervention and Erastin intervention were collected, total tissue RNA was extracted using the FastPure Cell RNA Isolation Kit V2 (Vazyme Cat. RC112.01) kit, and total RNA was reverse transcribed into cDNA using the HiScript III RT SuperMix for qPCR (+gDNA wiper) instructions from Vazyme and amplified on a PCR amplifier. The primers were designed and synthesized by Bioengineering (Shanghai) Co., Ltd. and the sequences are shown in **Table 3**. qPCR reaction total system: 0.4 μl each for upstream and downstream primers, 10.0 μl for Mix, 2.0 μl for cDNA, add ddH_2_O to 20.0 μl. reaction conditions: 95 °C 30 sec, 1 cycle; 95 °C 10sec, 60 °C 30 sec, 40 cycles; 95 The reaction conditions: 95 °C 15sec, 60 °C 60sec, 95 °C 15sec, 1 cycle. The relative expression of target genes was calculated using the 2-△△CT method. All the experiments were repeated six times with similar results.

**Table 3:** The primers for selected genes.

Gene	Forward primer	Reverse primer
RCC2	5’-CACGCAGAGCAGAAGGATGAGATG-3’	5’-CCCACTTCACTGACAGCAAAGGAG-3’
ABHD17B	5’-CTATGTTGCCTCTTCTGCTGTCCAC-3’	5’-ACAGATGTAAAGTCCAACGGCTTCC-3’
FTH1	5’-CTCCTACGTTTACCTGTCCATG-3’	5’-CAAGTCATCAGGCACATACAAG -3’
IRAK1	5’-ACGCTGACCTGGAGTGGACTG-3’	5’-GAAGCCGTTCTGAGCACAGTAGC-3’
GPX4	5’-ATGGTTAACCTGGACAAGTACC-3	5’-GACGAGCTGAGTGTAGTTTACT-3
β-Actin	5’-CCTGGCACCCAGCACAAT -3’	5’-GGGCCGGACTCGTCATAC-3’

### 2.10 Statistical analysis

*In vitro* experiments of our AML cell lines were performed using one-way ANOVA statistical methods for data analysis. Two independent samples t-test was used for PCR data of AML patients collected from hospitals.

## 3 Result

### 3.1 Clustering of co-expression modules Eigengenes in AML

**Figure 1:**
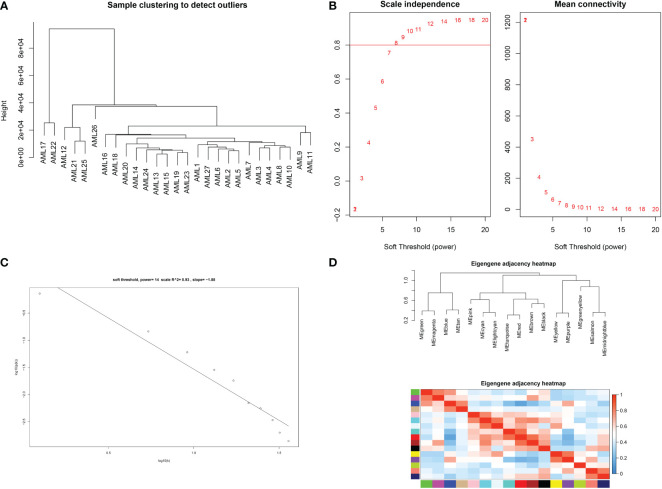
Determination of soft threshold parameters in WGCNA: **(A)** analysis of hospital sample outliers; **(B)** analysis of scale-free fit indices at different soft threshold β parameters and determination of the average connectivity at soft threshold parameters; **(C)** Correlation of log (k) and log [P(k)]; **(D)** sample clusters of module eigengenes..

The expression profiles of 27 samples from three risk class groups were included in the WGCNA. We used genes with the 5000 highest MAD values in the hospital dataset for further WGCNA. No discrete samples were identified from sample clustering (**Figure 1A**). To guarantee that the network was scale-free, we performed an empirical analysis to select the optimal β parameter. As shown in **Figures 1B, C**, the scale-free topological model fit index and the average connectivity reached a stable state when β was equal to 14. **Figure 1D** shows the clusters of module eigengenes.

### 3.2 Identification of key modules for AML

**Figure 2:**
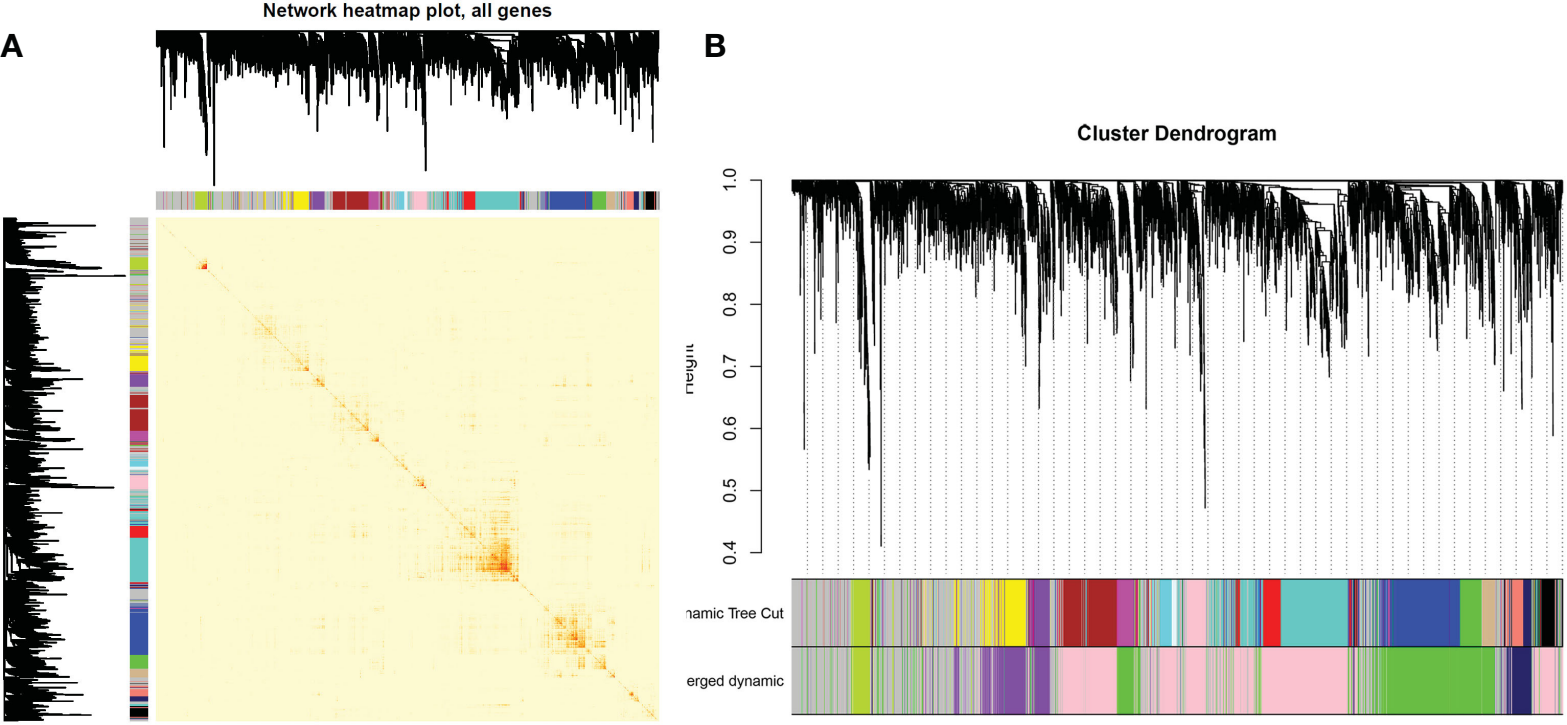
**(A)** Heat map of gene network visualization. The darker the shade of red, the better the overlap. **(B)** Dendrogram of all the differentially expressed genes based on clustering by the degree of difference (1-TOM).

**Figure 3:**
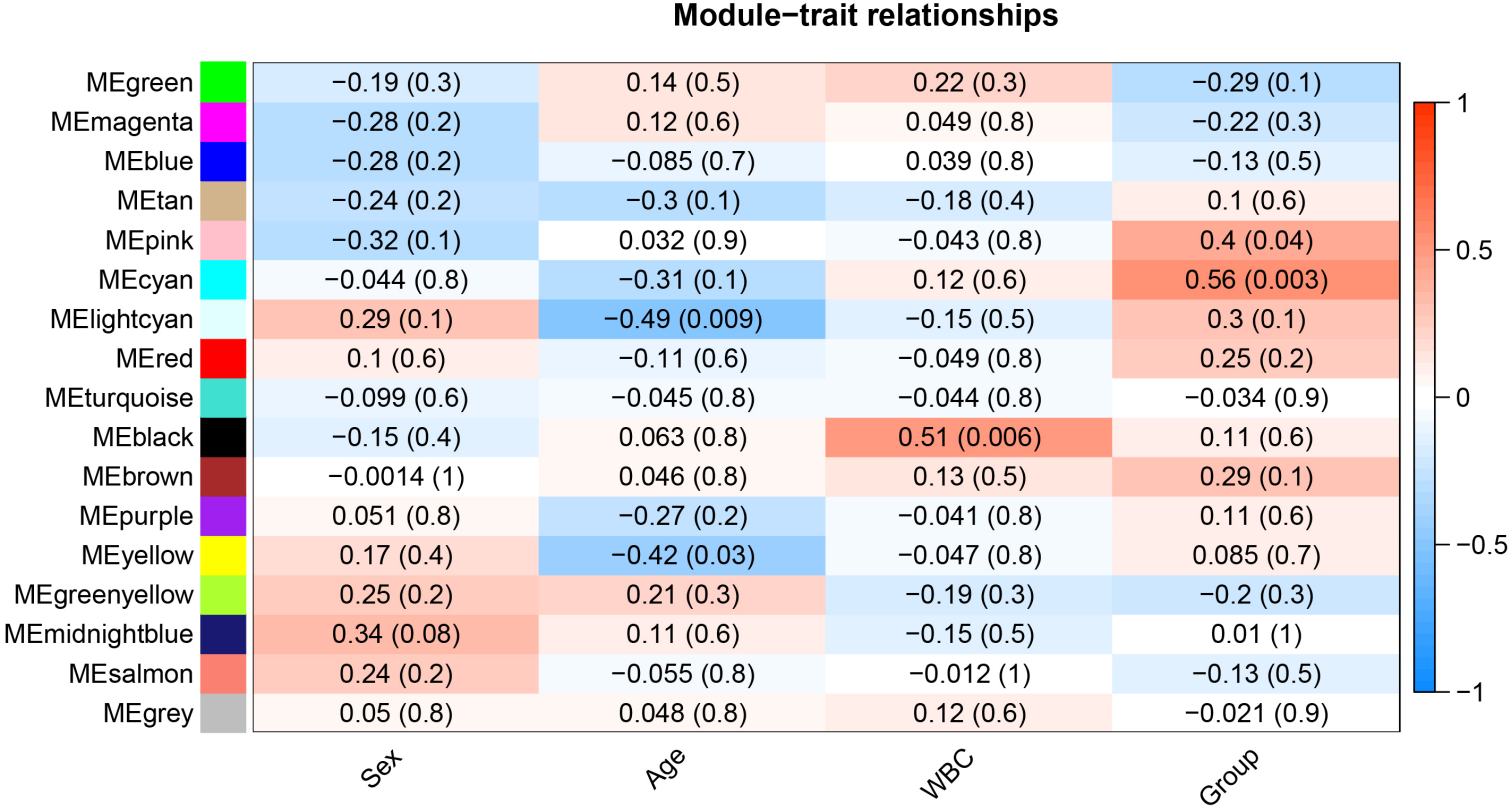
Heat map of correlation between different modules and clinical traits in AML.

After determining weighting factors, a dissTOM of 5000 genes was obtained (**Figure 2A**), and 17 modules were identified by mean linkage hierarchical clustering, each represented by a different color (**Figure 2B**). To explore the correlation between module feature values and different clinical traits, a heat map (**Figure 3**), of the 17 modules and the traits (sex, age, WBC and prognostic risk classification) was drawn. Children with AML were classified into three risk groups: favorable, intermediate, and poor.

The risk stratification was based in part on the current Chinese classification of cytogenetic and molecular genetic alterations in children with AML at initial diagnosis and the response of children to treatment, as well as on serological results (20). The risk stratification of the National Comprehensive Cancer Network was also used (21). Each column in **Figure 3** shows the correlation coefficient and the corresponding p-value. Red and blue represent positive and negative correlations respectively. The darker the color, the larger the correlation coefficient. We detected that each clinical trait was strongly correlated with a specific module, with the AML clinical risk class groupings most correlated with the pink and cyan modules. The correlation coefficients were 0.4 for the pink module (*p*= 0.04) and 0.56 for the cyan module(*p*=0.003). Therefore, these were selected for further analysis as clinically significant modules.

### 3.3 Identification of genes that are possibly significantly associated with AML in poor-risk groups

**Figure 4:**
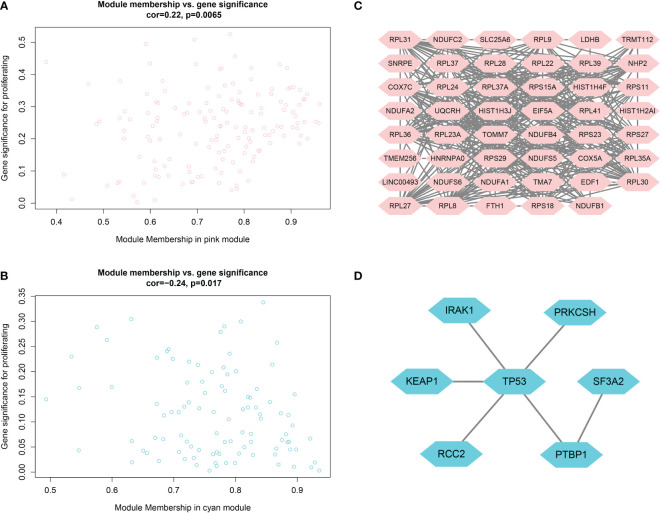
**(A, B)** Scatter plots of data concentration and p-value Cox regression. Each circle represents a gene. The X-axis indicates the degree of regression, the y-axis indicates the statistical significance of a gene. Network plots of key genes in the pink module; nodes indicate genes. **Figure 4** **(C, D)** PPI network diagram.

As shown in **Figures 4A, B**, candidate biomarker genes were selected based on the threshold values |MM|> 0.79 and |GS|> 0.2. |MM|> 0.79 indicated that the gene was associated with the module, whereas |GS|> 0.20 indicated that the gene expression profile was also phenotypically related. Finally, the linkage of each gene was the sum of the side attributes of the genes linked to it. The higher the degree of connectivity, the stronger the biological function of that gene. We obtained 53 candidate genes from the pink module and seven candidate genes from the cyan module. PPI networks covering the candidate genes were constructed using Cytoscape based on the PPI interactions from STRING (**Figures 4C, D**).

**Figure 5:**
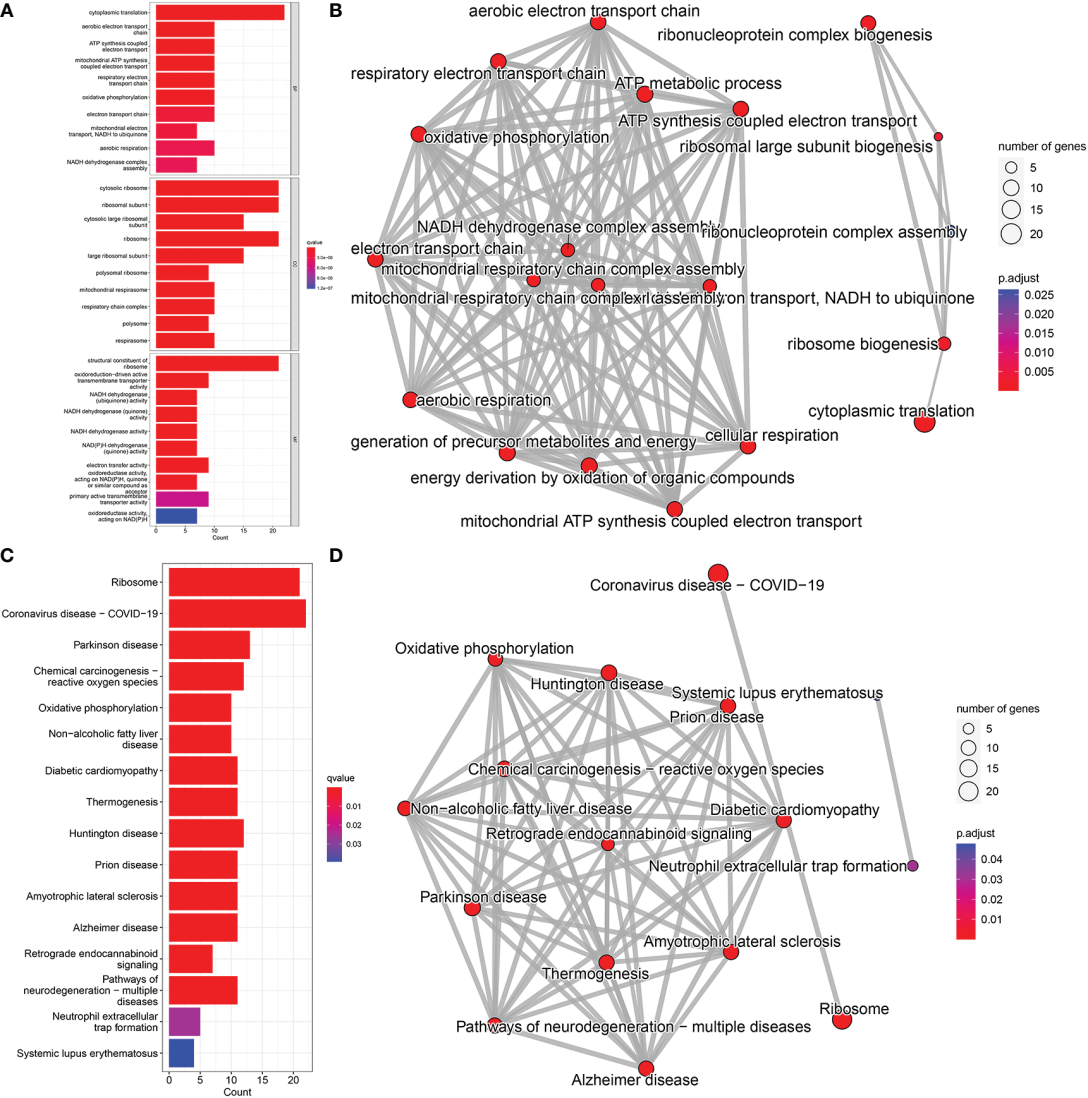
GO and KEGG pathway enrichment analysis; **(A)** BP, Biological Process; CC, Cellular Component; MF, Molecular Function. **(B)** Network diagram of GO analysis; **(C)** KEGG pathway analysis. **(D)** Network diagram of KEGG pathway analysis.

GO analysis revealed that candidate biomarker genes were mainly enriched biological processes annotated as cytoplasmic translation, aerobic electron transport chain, ATP synthesis coupled electron transport, and oxidative phosphorylation (**Figure 5A**) (22). Enrichment was mainly in molecular function annotations of structural components of ribosome and NADH dehydrogenase (ubiquinone) activity (23). The cellular components of the genes were significantly enriched in the annotations of cytosolic ribosome, cytosolic large ribosomal subunit, mitochondrial respirasome, respiratory chain complex (24). In addition, KEGG analysis showed that the candidate genes were mainly enriched in oxidative phosphorylation, and chemical carcinogenesis-reactive oxygen species pathways (**Figure 5C**) (25, 26).

### 3.4 Identification of biomarkers that predict poor prognosis in risk groups

**Figure 6:**
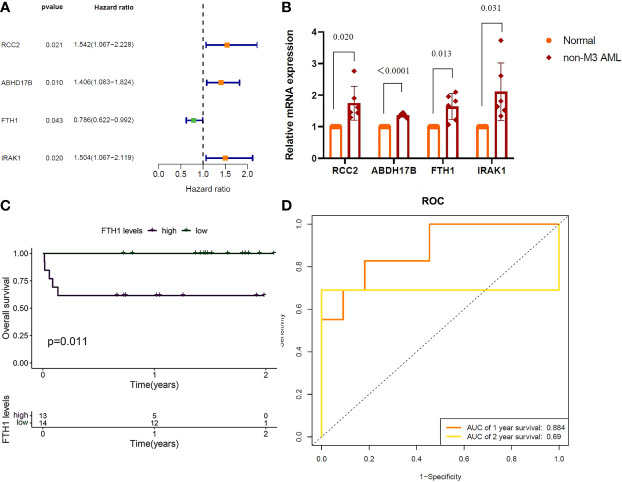
**(A)** *RCC2*: (*P*=0.021), Hazard Ratio (HR)=1.542; *ABHD17B*: (*P*= 0.010), R= 1.406. *FTH1*: (*P*=0.043), HR=0.786; *IRAK1*: (*P*=0.020), HR=1.504. **(B)** QRTPCR validation of RCC2, ABHD17B, FTH1 and IRAK1 expression, Normal versus AML (P=0.020, P < 0.0001, P=0.013, P=0.031). **(C)** Survival analysis of *FTH1* in hospital samples (purple represents that the gene is up in the sample and green represents that the gene is lowly expressed in the sample). **(D)** *FTH1*: 1-year AUC area of 0.953 and 2-year AUC area under the curve of 1.

Results of one-way Cox analysis in the TCGA database showed that four genes, *RCC2, ABHD17B, FTH1, IRAK1*, were associated with AML prognosis (**Figure 6A**). Compared with the normal group, the expression levels of RCC2, ABHD17B, FTH1 and IRAK1 were up-regulated in the AML group (**Figure 6B**). In the 27 hospital samples, patients were divided —as per median expression levels of the four candidate genes — into high and low expression groups. After survival analysis, *FTH1* was identified as a key gene for AML prognosis (**Figure 6C**). The specificity and sensitivity ROC were analyzed and the area under the curve (AUC) of the *FTH1* survival curve was calculated (**Figure 6D**).

**Figure 7:**
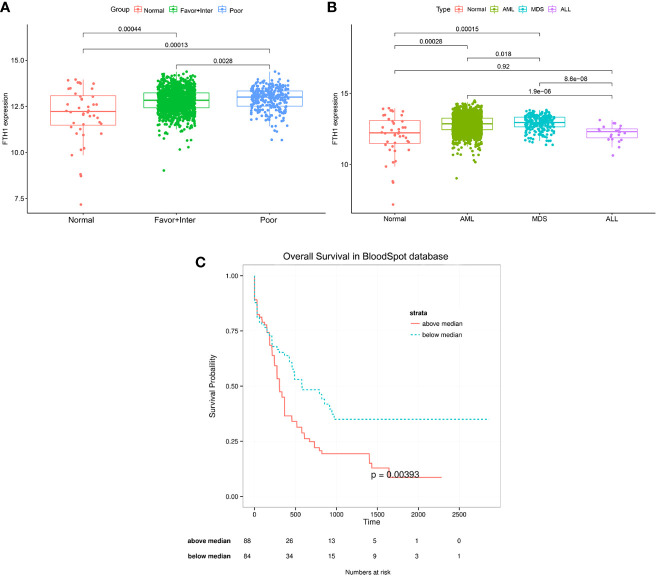
**(A, B)** Expression of *FHT1* in the normal group, risk class group, and statistical differences between the three groups. Normal: including Hematopoietic stem cell, Multipotential progenitors, Common myeloid progenitor cell, Granulocyte monocyte progenitors, Megakaryocyte-erythroid progenitor cell, Early Promyelocyte, Late Promyelocyte, Band cell, Metamyelocytes, Myelocyte, Monocytes, Polymorphonuclear cells; Favor-Inter:including AML with Normal karyotype, AML with inv (16), AML with t (15,17), AML with t (8,21), AML with Trisomy 8,AML with +7, AML with trisomy 11, AML with trisomy 13, AML with +7, AML with t (9,11);Poor: including AML with Complex karyotype, AML with t(11q23)/MLL, AML with del(5q), AML with del(7q)/7q-, AML with Complex del(5q),AML with Complex untypical karyotype, AML with inv (3),AML with t(6;9), AML with t(8;16), AML with t(1;3),AML with -5/7(q), AML with t(9;22);MDS: myelodysplastic syndrome, ALL: acute lymphoblastic leukemia. **Figure 7C**: The median value of FHT1 expression was taken for the grouping to compare the survival difference between the two groups, the group with greater than the median value of FHT1 had a significantly lower survival rate than the group with less than the median value of FHT1, the *p*-value was 0.00393.

The AML-related data samples were further validated by querying the public BloodSpot database (www.bloodspot.eu). From the expression levels of *FHT1* in normal and AML cells (**Figure 7A**), it was clear that levels in the poor-risk group were higher than those in normal cells (*p* = 0.00013) and low-medium risk group (*P* =0.0028). From the expression levels of *FTH1* in normal, AML, MDS, and ALL cells, levels in AML leukemia cells were increasingly higher than those in normal cells (*P*=0.00028) (**Figure 7B**). The survival curves with very differences in survival rates between the two groups were separated into poor and favorable groups according to the median value of *FTH1* expression levels (**Figure 7C**).

### 3.5 CCK8 assays validate MOLM-13 and THP-1 proliferation

**Figure 8:**
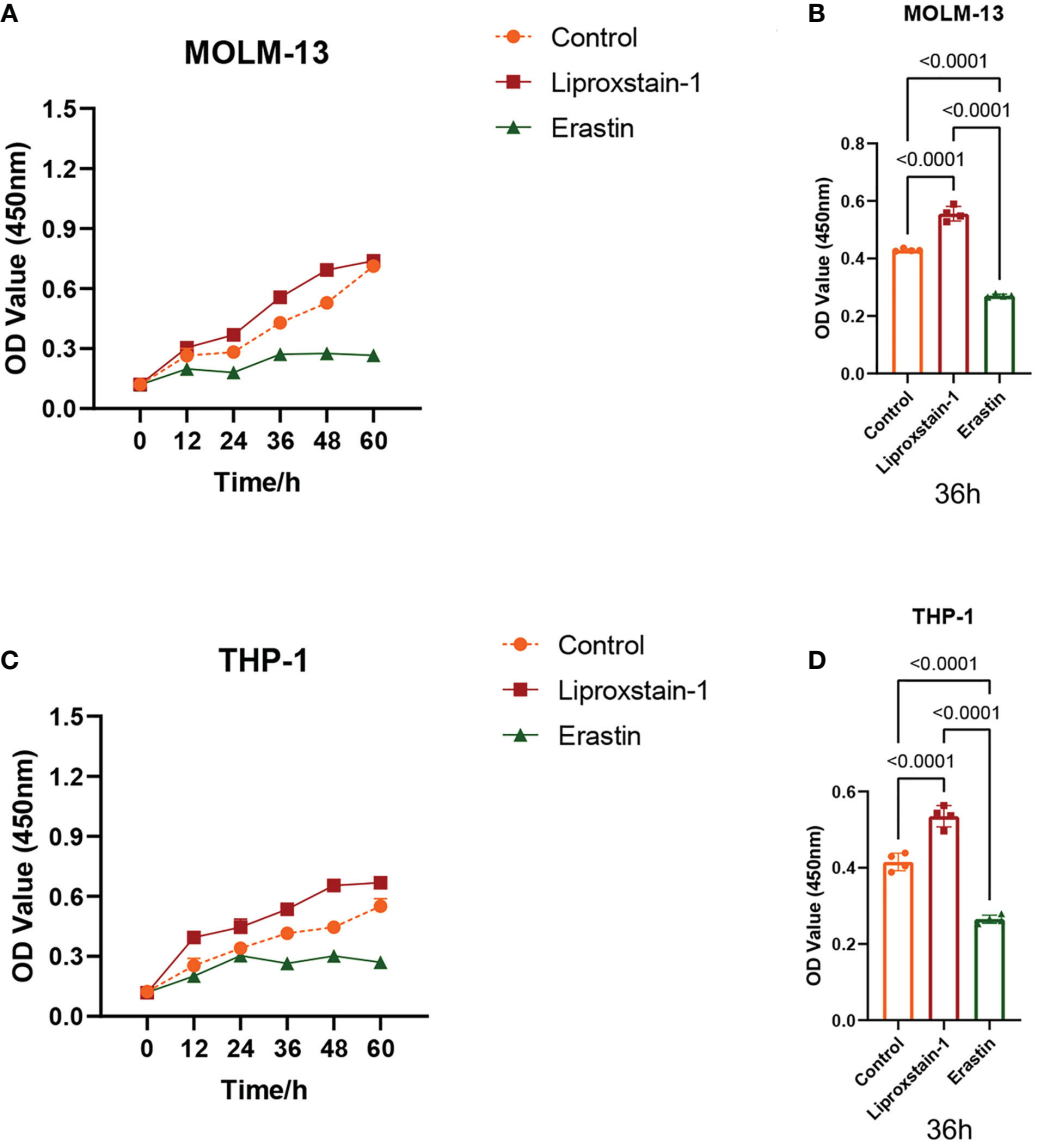
**(A, C)** The OD value of MOLM-13 and THP-1. **(B, D)** Comparison of OD values of Liproxstain-1 group, Erastin group, and control group at 36 hours.

OD values were positively correlated with AML cell proliferation (**Figures 8A, C**). The OD value of Liproxstain-1 group was higher than that of the control (without drug intervention) at 36 hours post-inoculation, (*P*<0.0001). Conversely, the OD of the Erastin group was lower than that of the control (*P*<0.0001) (**Figures 8B, D**) confirming that ferroptosis processes affect proliferation in leukemic cells.

### 3.6 Cell apoptosis

**Figure 9:**
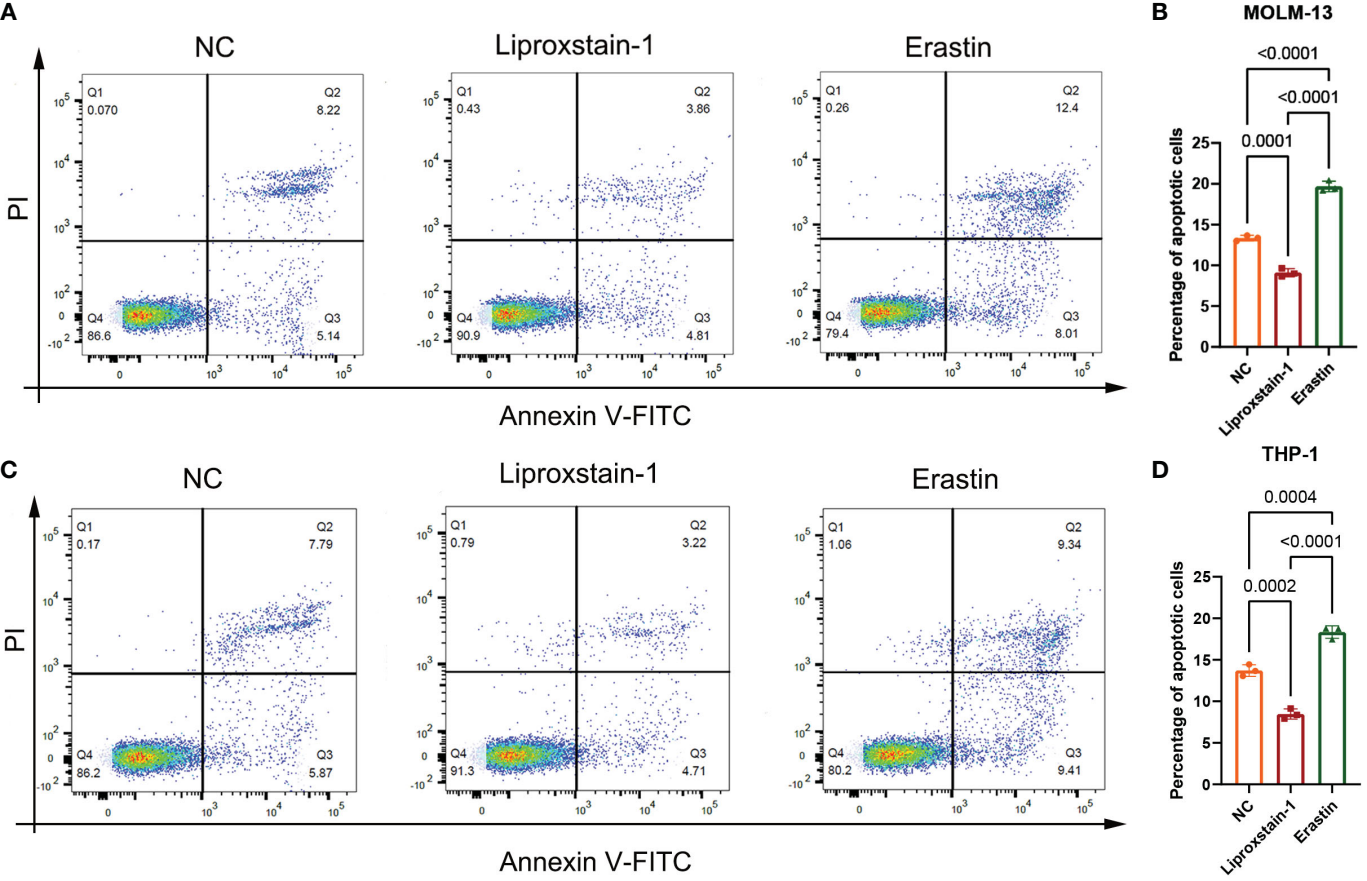
**(A, B)** MOLM-13. **(C, D)** THP-1. NC: Negative control group flow cytometry, apoptosis accounted for 13.36 and 13.66, respectively. flow cytometry of liproxstain-1 group showed that the proportion of apoptosis was 8.67 and 7.93, respectively. Erastin group flow cytometry, apoptosis accounted for 20.41 and 18.75, respectively. **(B)** Histogram of apoptosis rate of MOLM-13 detected by flow cytometry. **(D)** Histogram of apoptosis rate of THP-1 detected by flow cytometry.

Flow cytometry results, as shown in **Figure 9**, showed that there was a statistical difference (*P*<0.05) between the increased apoptosis of MOLM-13 and THP-1 cells in the Erastin group (*P*<0.001) and the decreased apoptosis of MOLM-13 and THP-1 cells in the Liproxstain-1 group (*P*<0.001) compared to the negative control group, suggesting that the ferroptosis process of leukemic cells can influence the occurrence of apoptosis.

### 3.7 QRT-PCR validation of *FTH1* and *GPX4* expression

**Figure 10:**
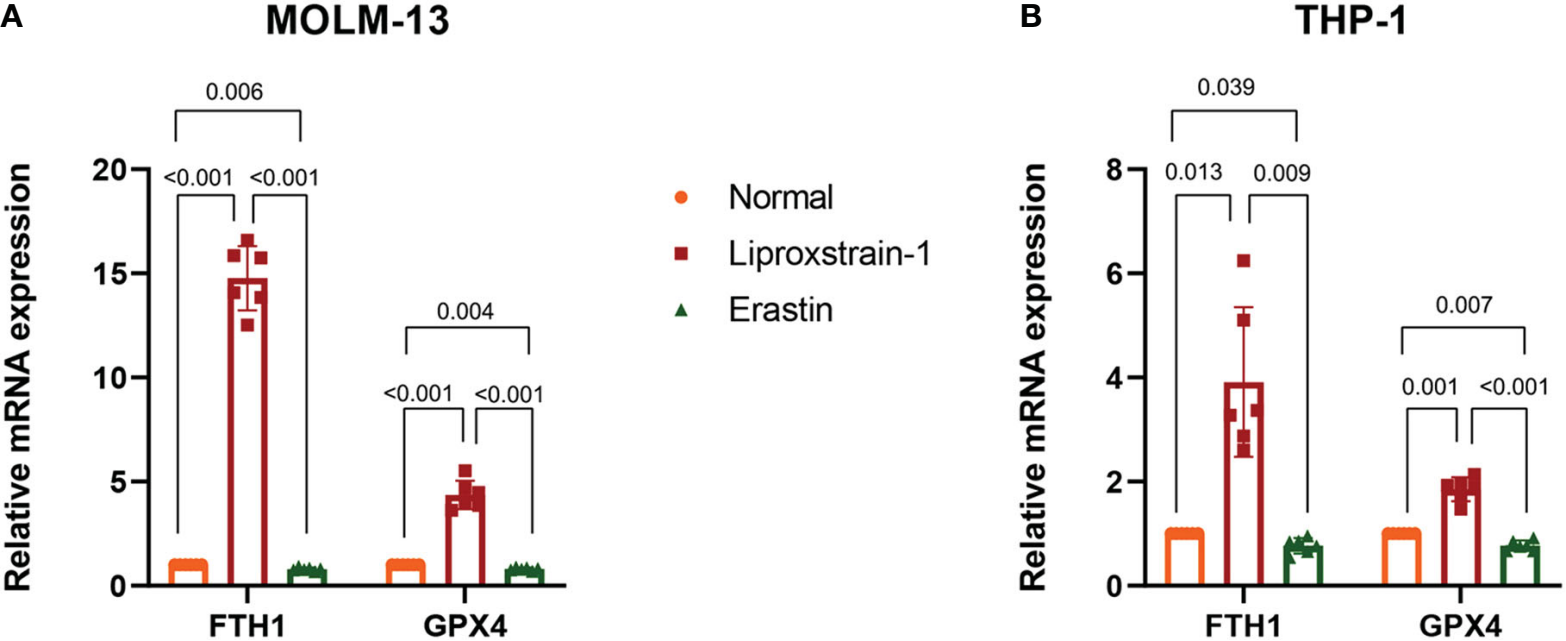
**(A)** QRT-PCR validation of *FTH1* and *GPX4* expression. Normal versus Liproxstain-1, (*P*<0.001); Normal versus Erastin, (*P*=0.006, *P*=0.004); Liproxstain-1 versus Erastin, (*P*<0.001). **(B)** QRT-PCR validation of *FTH1* and *GPX4* expression. Normal versus Liproxstain-1, (*P*=0.013, *P*=0.001); Normal versus Erastin, (*P*=0.039, *P*=0.007); Liproxstain-1 versus Erastin, (*P*=0.009, *P*<0.001).

To investigate the expression of *FTH1* and *GPX4* after ferroptosis inhibitor and ferroptosis agonist intervention on MOLM-13 and THP-1 cells, we performed a qRT-PCR analysis. *FTH1* and *GPX4* expression was statistically significantly upregulated and downregulated in the Liproxstain-1 and Erastin groups respectively (**Figures 10A, B**).

## 4 Discussion

AML is a malignant disease of hematopoietic stem cells characterized by clonal expansion of abnormally differentiated primitive cells in the bone marrow spectrum, and both AML patient-related and disease-related factors affect the likelihood of achieving therapeutic response and long-term survival in individual patients (27).

Using WGCNA we identified 60 genes, not only involved in constituting the oxidoreductase complex but also enriched in the oxidative phosphorylation, chemoattractive- reactive oxygen pathways. AML cells have an atypical metabolic phenotype characterized by increased mitochondrial mass and greater dependence on oxidative phosphorylation and fatty acid oxidation. Alterations in these genes lead to abnormalities in oxidative phosphorylation of the organism, and the extent of lipid peroxidation product accumulation that is regulated by lipid peroxide production and clearance possibly deters the onset of ferroptosis (28). Indeed, Zhang and colleagues suggested that PKCβII-mediated phosphorylation of ACSL4 activated ACSL4, which then promoted the production of PUFA-containing phospholipids, leading to ferroptosis (29). We hypothesize that these candidate genes may be involved in the onset and progression of AML disease through phosphorylation signaling pathways, with associated prognostic implications.

**Figure 11:**
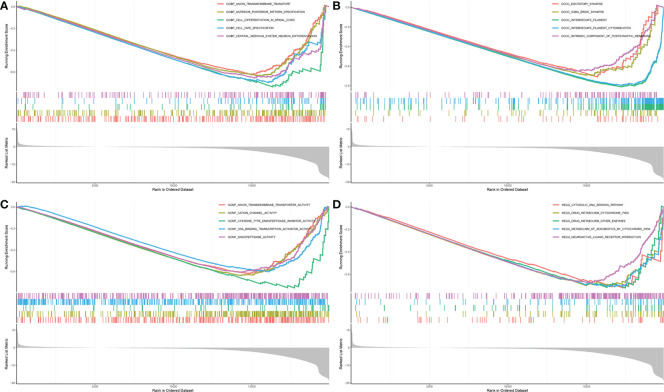
**(A)** biological process; **(B)** cellular component; **(C)** biological process; **(D)** KEGG.

One-way Cox analysis was performed on the 60 candidate genes using the TCGA dataset, and the *FTH1* gene was identified by survival analysis as a key gene. Based on median expression levels of *FTH1*, patients were divided into high and low *FTH1* expression groups. Patients with high *FTH1* expression had higher overall mortalities compared to those with low *FTH1* expressions. Thus, *FTH1* gene set enrichment analysis was performed using GO terms for biological processes, molecular functions, and cellular components. For biological processes, enriched annotations were anion transmembrane transport (30) and cell differentiation in the spinal cord (31) (**Figure 11A**). For cellular components, enriched annotations were associated with intermediate filament cytoskeleton (**Figure 11B**). For molecular functions, enriched annotations were associated with anion transmembrane transport cation channel and endopeptidase activities (**Figure 11C**). KEGG enrichment analysis indicated that FTH1 overexpression may be mediated through the cytosolic DNA sensing (32) and drug metabolism cytochrome P450 (33) pathways.

*FTH1* encodes the heavy subunit of ferritin, whose obvious iron oxidase activity, due to glutamate residues, is helpful for the rapid uptake of iron as a metal ligand (34, 35). *FTH1* is closely associated with malignant tumors such as breast and liver cancers (36, 37) as well as hematological malignancies (38). *FTH1* plays an anti-cancer role by promoting angiogenesis (39, 40). Increased expression of *FTH1* inhibits cancer by promoting apoptosis (41).

**Figure 12:**
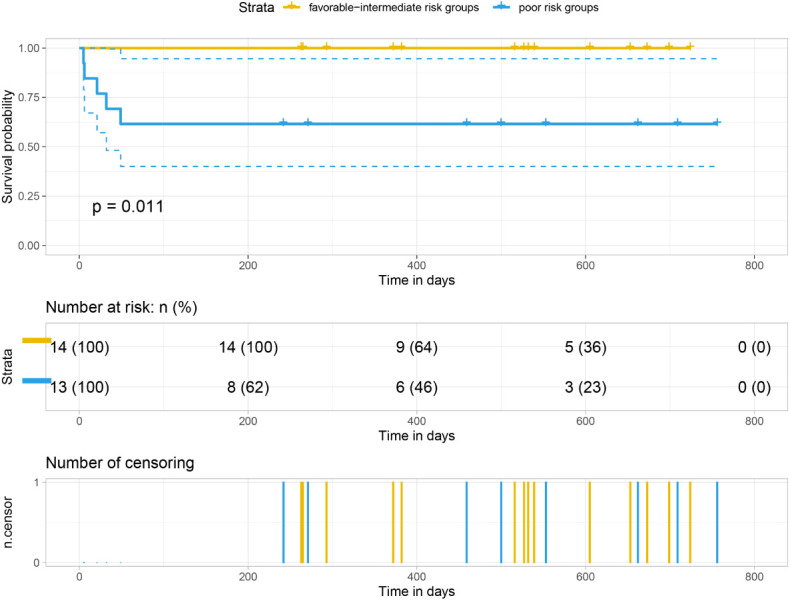
Survival curves of 27 pediatric AML patients.

**Figure 13:**
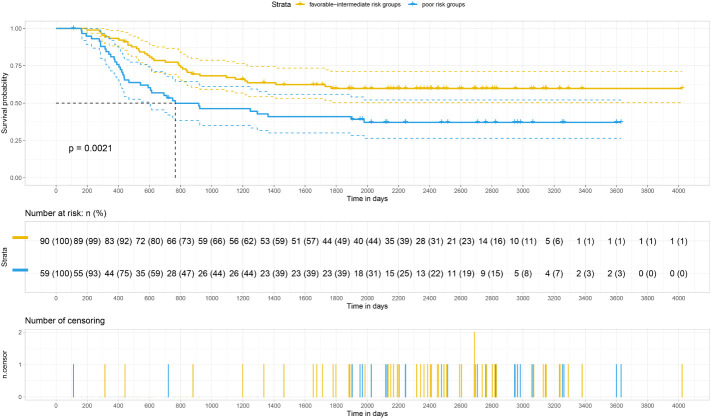
Survival curves of AML in 149 children from the TCGA database.

Our analysis of the data obtained from the BloodSpot database showed that mRNA expression level of *FTH1* was highest in the high-risk group. Moreover, expression levels differed among blood diseases with AML having higher *FTH1* expression levels than both ALL and normal human cells (42). The high expression of *FTH1* was positively correlated with mortality rates in AML patients. These results demonstrated the prognostic potential of the expression level of *FTH1*. Indeed, the survival rate of patients in the high-risk group was verified to be lower than that of patients in the low- (*P*=0.011) and intermediate-risk groups (*P*=0.0021) as per survival analysis of the hospital and TCGA data sets (**Figures 12**, **13**) confirming the effect *FTH1* expression level has on the prognostic risk class grouping. Thus, we hypothesize that high *FTH1* expression level is a risk factor for AML in children. This possibly provides new strategies for AML treatment in children.

The ferroptosis is impaired by lipid oxide metabolism in cells, and GPX4 can degrade small molecules of hydrogen peroxide and some lipid oxides (12). In the presence of active iron, it catalyzes the production of specific phospholipid hydroperoxides, and this chemical process can be counteracted by endogenous GPX4, thus acting as an inhibitor of ferroptosis (43, 44). Overexpression of *FTH1* suppresses the progression of ferroptosis (45). In rat leukemia cells, overexpression of GPX4 inhibits Cytochrome c release, caspase activation, NFB activation, and DNA cleavage (46). In this study, overexpression of *FTH1* inhibited ferroptosis and thus contributed to the proliferation of leukemic cells, enhancing the sensitivity of AML cells to chemotherapeutic agents by the ferroptosis inducer erastin. Iron overload in AML patients may lead to a variety of cellular and systemic changes and therefore plays a critical role in these hematologic malignancies. In our cell proliferation assay, MOLM-13 and THP-1 cells treated with Liproxstain-1 proliferated much more than the controls. Conversely, cells treated with erastin, a ferroptosis inducer that inhibits the cysteine glutamate reverse transport system, decrease cysteine input and subsequent glutathione synthesis, and have restricted cell proliferation. In AML cell lines, in a dose-dependent manner, erastin affects mixed cell death including ferroptosis, and enhances the anti-leukemic effects of cytarabine and doxorubicin (47).

Thus, our findings suggest that the ferroptosis process affects the proliferative status of leukemic cells, consistent with the finding that early cell death in AML is associated with ferroptosis induction (48). We further analyzed the expression level of *FHT1* in MOLM-13 and THP-1 cells by qPCR and found that *FTH1* was significantly upregulated when MOLM-13 and THP-1 cells were actively proliferating, and downregulated when MOLM-13 and THP-1 cell proliferation was impaired. Furthermore, leukemic cells in the AML high-risk group had high expression levels of *FTH1* and actively proliferated. Therefore, we inferred that overexpression of *FTH1* may be one of the risk factors for AML in children. By inhibiting the *FTH1* expression, the proliferation of AML leukemia cells could be negatively affected, resulting in a better prognosis of childhood non-M3 AML. But our inference needs more experiments to prove.

## 5 Conclusion

We identified *FTH1* as a key gene that affects the risk of a poor prognosis, thus establishing a possible tool for improving the prognosis of AML patients in the clinical setting.

## Data availability statement

The datasets presented in this study can be found in online repositories. The names of the repository/repositories and accession number(s) can be found in the article/supplementary material.

## Ethics statement

Ethical approval was granted by medical ethics committee of Affiliated Children’s Hospital of Suzhou University and the local ethics committees of The First Affiliated Hospital of Guangxi Medical University in Guangxi Province, China, with written informed consent obtained from all participants. (No. 2017047-1). Written informed consent to participate in this study was provided by the participants’ legal guardian/next of kin. Written informed consent was obtained from the individual(s), and minor(s)’ legal guardian/next of kin, for the publication of any potentially identifiable images or data included in this article.

## Author contributions

YH recruited patients and conducted clinical evaluations; YH designed the study, and searched for funding; JZ drafted the manuscript and carried out statistical and bioinformatic analyses; LL and JW participated in TGCA sample collection and clinical analyses; JZ wrote the final version of the paper. All authors contributed to the article and approved the submitted version.
