# Corrigendum: High expression level of the FTH1 gene is associated with poor prognosis in children with non-M3 acute myeloid leukemia

**DOI:** 10.3389/fonc.2023.1212991

**Published:** 2023-08-21

**Authors:** Junlin Zhang, Liying Liu, Jinshuang Wei, Xiaojing Wu, Jianming Luo, Hongying Wei, Liao Ning, Yunyan He

**Affiliations:** ^1^ First Affiliated Hospital of Guangxi Medical University, Nanning, China; ^2^ The Key Laboratory of Children’s Disease Research in Guangxi’s Colleges and Universities, Education Department of Guangxi Zhuang Autonomous Region, Nanning, China

**Keywords:** AML, WGCNA, FTH1, ferroptosis, prognosis

In the published article, there was an error in affiliations 1,2,3. Instead of “1. First Affiliated Hospital of Guangxi Medical University, Nanning, China, 2. The Key Laboratory of Children’s Disease Research in Guangxi’s Colleges and Universities, Education Department of Guangxi Zhuang Autonomous Region, Nanning, China, 3. The Affiliated Children’s Hospital of Suzhou University, Suzhou, Jiangsu, China” it should be “1. First Affiliated Hospital of Guangxi Medical University, Nanning, China, 2. The Key Laboratory of Children’s Disease Research in Guangxi’s Colleges and Universities, Education Department of Guangxi Zhuang Autonomous Region, Nanning, China".

In the published article, there was an error in the legend for **Figure 6** as published. **Figure 6B** is missing statement describing PCR results. The corrected legend appears below.

**Figure 6:**
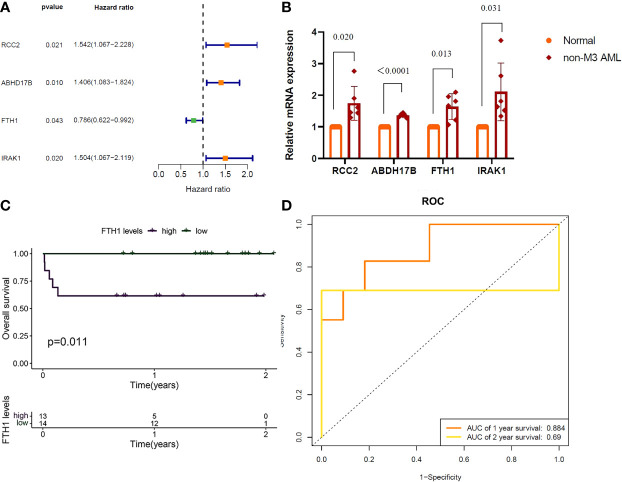
**(A)** RCC2: (P=0.021), Hazard Ratio (HR)=1.542; ABHD17B: (P=0.010), R=1.406. FTH1: (P=0.043), HR=0.786; IRAK1: (P=0.020), HR=1.504. **(B)** QRT-PCR validation of RCC2, ABHD17B, FTH1 and IRAK1 expression, Normal versus AML (P=0.020, P < 0.0001, P=0.013, P=0.031). **(C)** Survival analysis of FTH1 in hospital samples (purple represents that the gene is up in the sample and green represents that the gene is lowly expressed in the sample). **(D)** FTH1: 1-year AUC area of 0.953 and 2-year AUC area under the curve of 1.

**(A)** RCC2: (P=0.021), Hazard Ratio (HR)=1.542; ABHD17B: (P=0.010), R=1.406. FTH1: (P=0.043), HR=0.786; IRAK1: (P=0.020), HR=1.504. **(B)** QRT-PCR validation of RCC2, ABHD17B, FTH1 and IRAK1 expression, Normal versus AML (P=0.020, P < 0.0001, P=0.013, P=0.031). **(C)** Survival analysis of FTH1 in hospital samples (purple represents that the gene is up in the sample and green represents that the gene is lowly expressed in the sample). **(D)** FTH1: 1-year AUC area of 0.953 and 2-year AUC area under the curve of 1.

In the published article, there was an error in

**Table 3** as published. Reverse primer format for ABHD17B is incorrect. The corrected **Table 3** appears below.

**Table 3:** The primers for selected genes.

Gene	Forward primer	Reverse primer
RCC2	5’-CACGCAGAGCAGAAGGATGAGATG-3’	5’-CCCACTTCACTGACAGCAAAGGAG-3’
ABHD17B	5’-CTATGTTGCCTCTTCTGCTGTCCAC-3’	5’-ACAGATGTAAAGTCCAACGGCTTCC-3’
FTH1	5’-CTCCTACGTTTACCTGTCCATG-3’	5’-CAAGTCATCAGGCACATACAAG -3’
IRAK1	5’-ACGCTGACCTGGAGTGGACTG-3’	5’-GAAGCCGTTCTGAGCACAGTAGC-3’
GPX4	5’-ATGGTTAACCTGGACAAGTACC-3	5’-GACGAGCTGAGTGTAGTTTACT-3
β-Actin	5’-CCTGGCACCCAGCACAAT -3’	5’-GGGCCGGACTCGTCATAC-3’

In the published article, there was an error. **Figure 6B** lacks reference and description in the legend.

A correction has been made to “**3 Result”**, “*3.4 Identification of biomarkers that predict poor prognosis in risk groups* **”**, paragraph 1. This sentence previously stated:

“Results of one-way Cox analysis in the TCGA database showed that four genes, RCC2, ABHD17B, FTH1, IRAK1, were associated with AML prognosis (**Figure 6A**). In the 27 hospital samples, patients were divided —as per median expression levels of the four candidate genes— into high and low expression groups. After survival analysis, FTH1 was identified as a key gene for AML prognosis (**Figure 6B**). The specificity and sensitivity ROC were analyzed and the area under the curve (AUC) of the FTH1 survival curve was calculated (**Figure 6C**).”

The corrected sentence appears below:

“Results of one-way Cox analysis in the TCGA database showed that four genes, RCC2, ABHD17B, FTH1, IRAK1, were associated with AML prognosis (**Figure 6A**). Compared with the normal group, the expression levels of RCC2, ABHD17B, FTH1 and IRAK1 were up-regulated in the AML group (**Figure 6B**). In the 27 hospital samples, patients were divided —as per median expression levels of the four candidate genes— into high and low expression groups. After survival analysis, FTH1 was identified as a key gene for AML prognosis (**Figure 6C**). The specificity and sensitivity ROC were analyzed and the area under the curve (AUC) of the FTH1 survival curve was calculated (**Figure 6D**).”

The authors apologize for these errors and state that these do not change the scientific conclusions of the article in any way. The original article has been updated.

