# Supplementary figures and images for: High expression level of the FTH1 gene is associated with poor prognosis in children with non-M3 acute myeloid leukemia
Source: Front Oncol. 2023 Feb 1;12:1068094. doi: 10.3389/fonc.2022.1068094 (PMC9928996; doi:10.3389/fonc.2022.1068094)

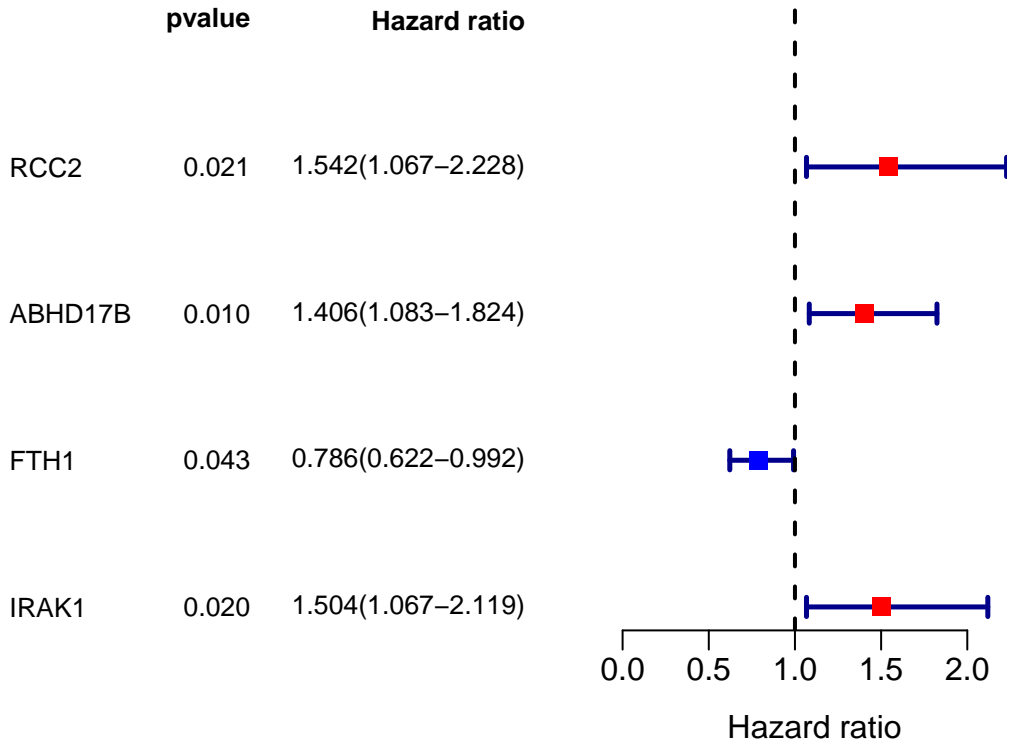

Supplement: Supplementary file 3 [file DataSheet_3.zip › cox/forest.pdf]

# FTH1

Type ■ Normal ■ AML ■ MDS ■ ALL

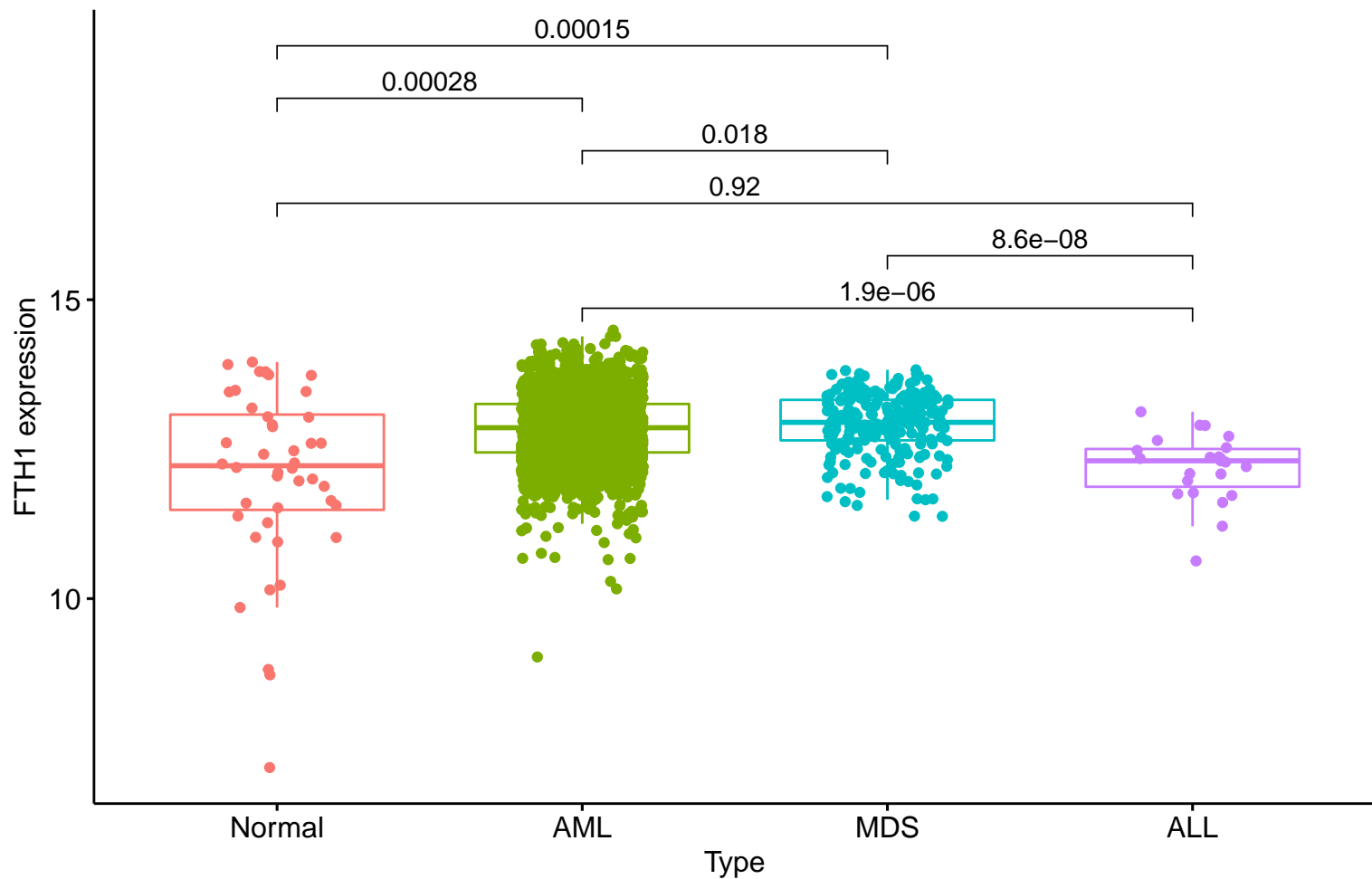

Supplement: Supplementary file 4 [file DataSheet_4.zip › FTH1 BloodSpot/Type..pdf]

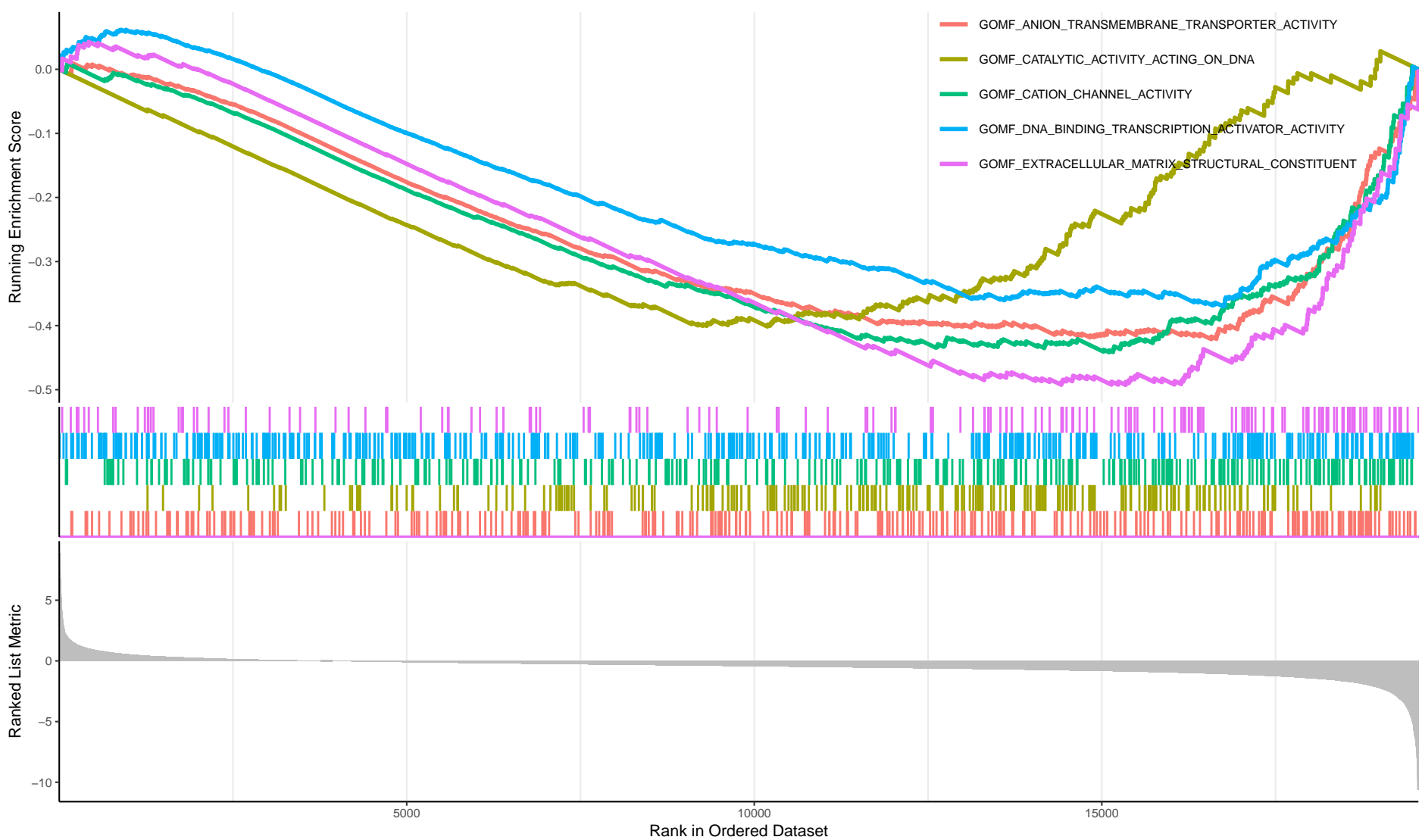

Supplement: Supplementary file 6 [file DataSheet_6.zip › GSEA/Termmf..pdf]

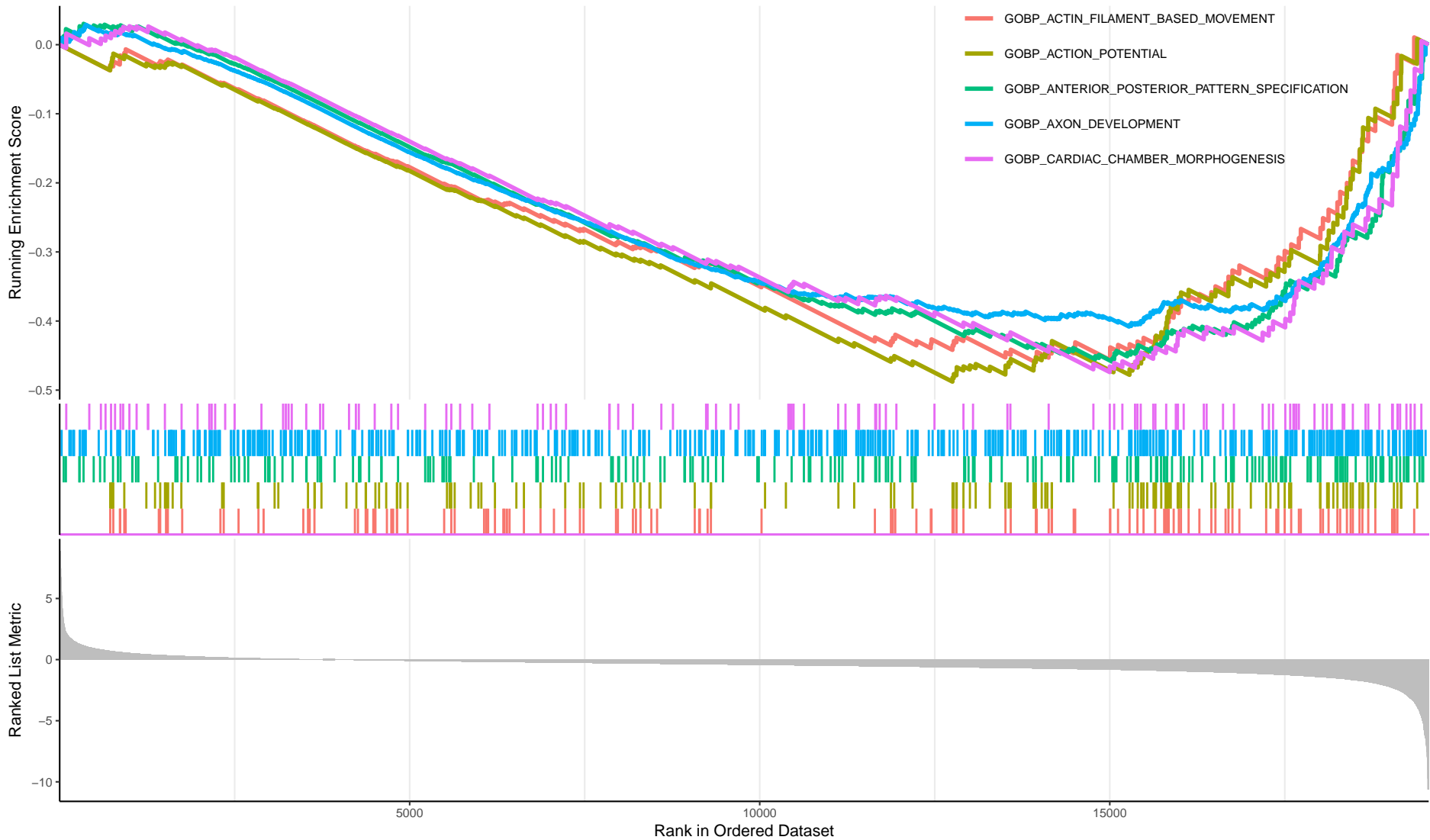

Supplement: Supplementary file 6 [file DataSheet_6.zip › GSEA/Termmfbp..pdf]

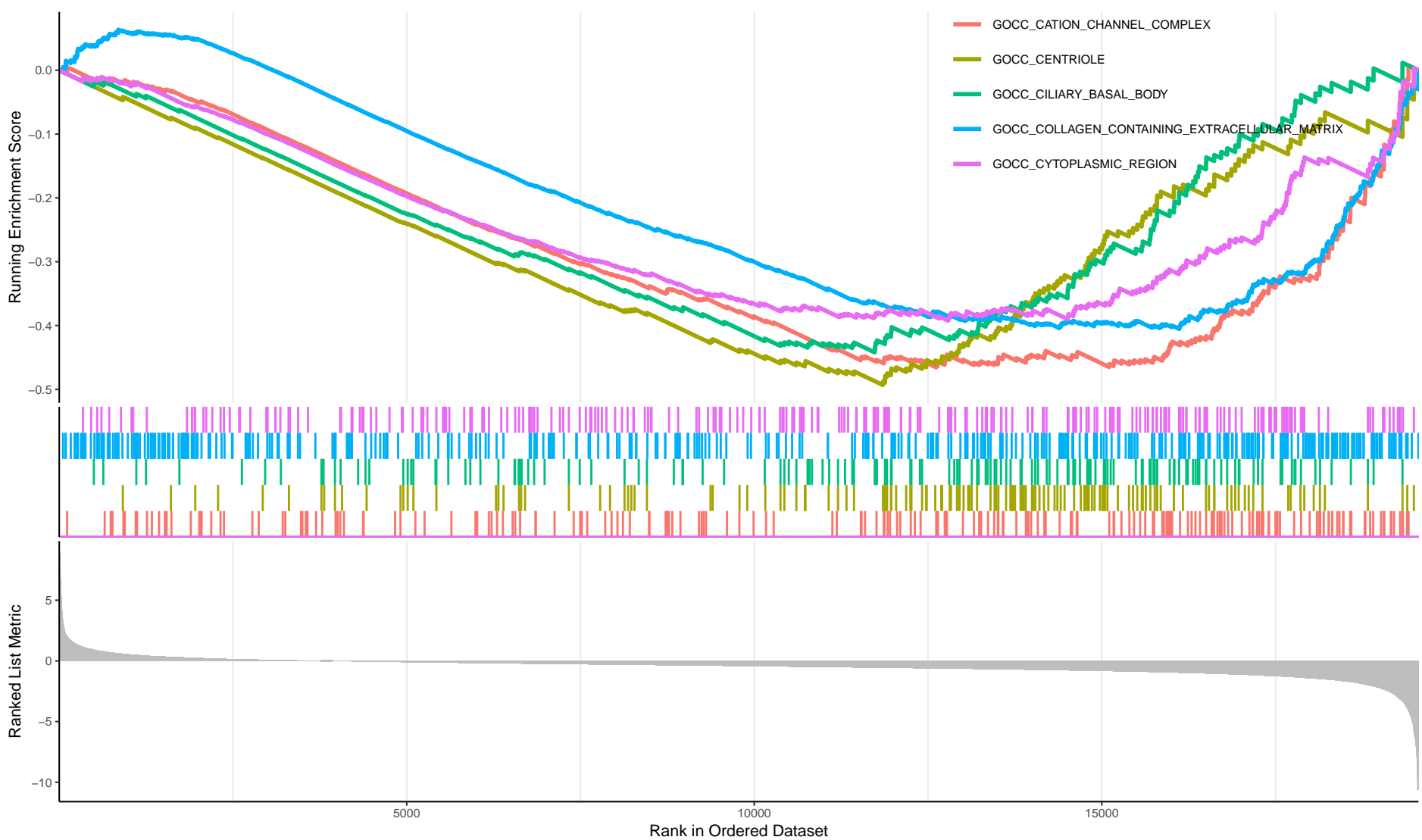

Supplement: Supplementary file 6 [file DataSheet_6.zip › GSEA/Termmfcc..pdf]
