# Supplementary material for: High expression level of the FTH1 gene is associated with poor prognosis in children with non-M3 acute myeloid leukemia
Source: Front Oncol. 2023 Feb 1;12:1068094. doi: 10.3389/fonc.2022.1068094 (PMC9928996; doi:10.3389/fonc.2022.1068094)
Supplement: Supplementary file 6 [file DataSheet_6.zip › GSEA/KEGG..pdf]

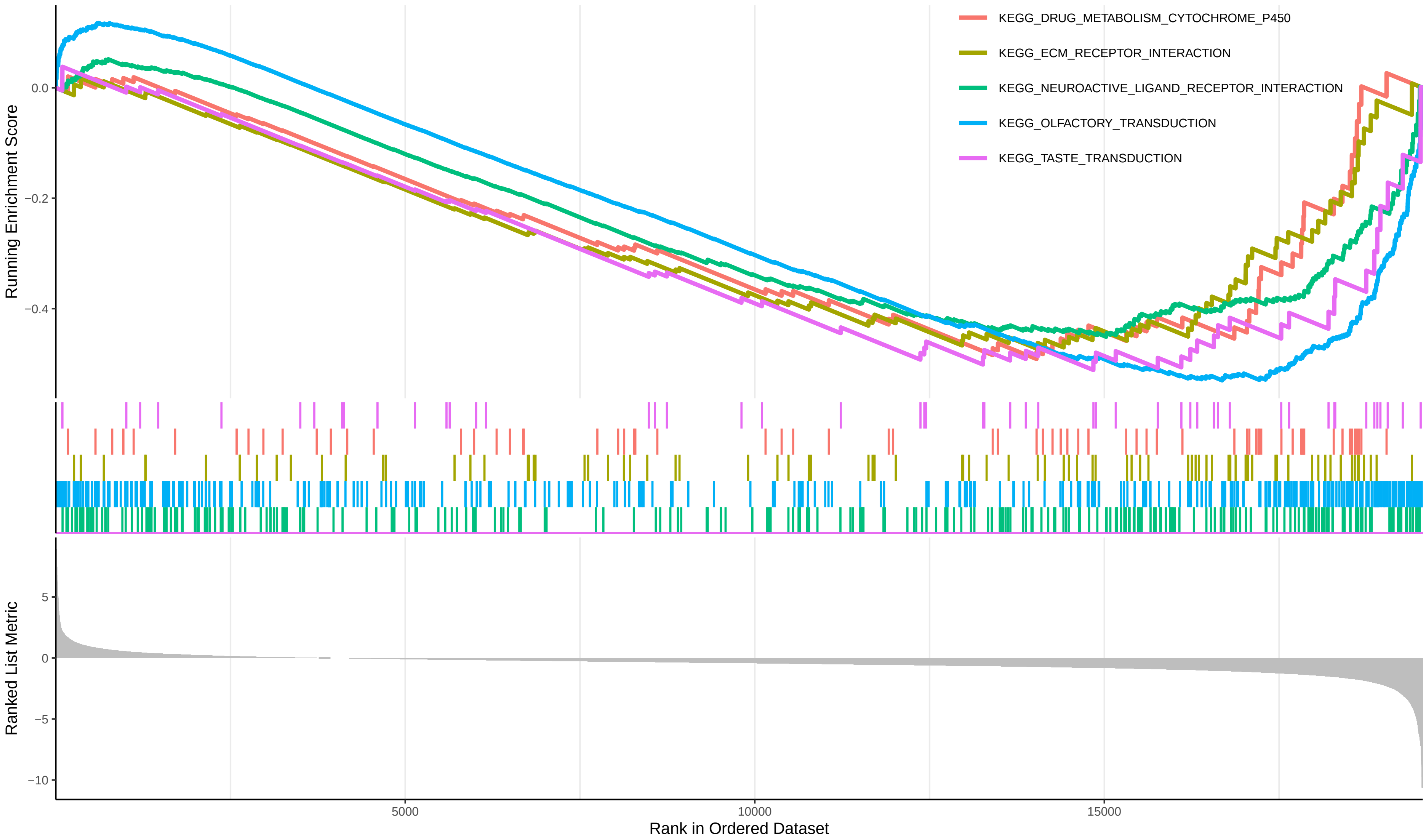

KEGG_DRUG_METABOLISM_CYTOCHROME_P450
KEGG_ECM_RECEPTOR_INTERACTION
KEGG_NEUROACTIVE_LIGAND_RECEPTOR_INTERACTION
KEGG_OLFACTORY_TRANSDUCTION
KEGG_TASTE_TRANSDUCTION
Running Enrichment Score
0.0
−0.2
−0.4
Ranked List Metric
5
0
−5
−10
5000
10000
15000
Rank in Ordered Dataset
